# Mutual Coupling Reduction through Defected Ground Structure in Circularly Polarized, Dielectric Resonator-Based MIMO Antennas for Sub-6 GHz 5G Applications

**DOI:** 10.3390/mi13071082

**Published:** 2022-07-08

**Authors:** Anwar Ali, Jijun Tong, Javed Iqbal, Usman Illahi, Abdul Rauf, Saeed Ur Rehman, Haider Ali, Muhammad Mansoor Qadir, Muhammad Amir Khan, Rania M. Ghoniem

**Affiliations:** 1 School of Information Science and Technology, Zhejiang Sci-Tech University, Hangzhou 310018, China; anwar.ali@zstu.edu.cn (A.A.); jijuntong@zstu.edu.cn (J.T.); 2 School of Electrical and Electronic Engineering, Engineering Campus, Universiti Sains Malaysia, Nibong Tebal, Penang 14300, Malaysia; usmanillahi83@gmail.com; 3 Electrical Engineering Department, Gomal University, Dera Ismail Khan 29050, Pakistan; 4 Department of Electrical Engineering, National University of Sciences and Technology, Islamabad 44000, Pakistan; a.rauf@nust.edu.pk; 5 Electrical Engineering Department, Attock Campus, Comsats University Islamabad, Abbottabad 22060, Pakistan; saeedurrehman@cuiatk.edu.pk; 6 Department of Electrical and Electronics Engineering Technology, University of Technology, Nowshera 24100, Pakistan; haider.ali@uotnowshera.edu.pk; 7 Department of Computer Science, Iqra National University, Peshawar 25000, Pakistan; mansoor@inu.edu.pk; 8 Department of Computer Science, Islamabad-Abbottabad Campus, COMSATS University, Abbottabad 22060, Pakistan; 9 Department of Information Technology, College of Computer and Information Sciences, Princess Nourah bint Abdulrahman University, P.O. Box 84428, Riyadh 11671, Saudi Arabia; rmghoniem@pnu.edu.sa

**Keywords:** singly-fed, circular polarization, dielectric resonator antenna (DRA), defected ground structure, multiple-input-multiple-output (MIMO), 5G NR band

## Abstract

This paper describes a singly-fed circularly polarized rectangular dielectric resonator antenna (RDRA) for MIMO and 5G Sub 6 GHz applications. Circular polarization was achieved for both ports using a novel-shaped conformal metal strip. To improve the isolation between the radiators, a “S” shaped defective ground plane structure (DGPS) was used. In order to authenticate the estimated findings, a prototype of the suggested radiator was built and tested experimentally. Over the desired band, i.e., 3.57–4.48 GHz, a fractional impedance bandwidth of roughly 36.63 percent (−10 dB as reference) was reached. Parallel axial ratio bandwidth of 28.33 percent is achieved, which is in conjunction with impedance matching bandwidth. Between the ports, isolation of −28 dB is achieved Gain and other far-field parameters are also calculated and found to be within their optimum limits

## 1. Introduction

Multiple antennas were used at the transmitter and reception sides of the multiple-input-multiple-output (MIMO) antenna system to improve system capability without requiring additional power. High separation among the radiating elements is needed to provide improved channel capacity. Because of the absence of side waves, as well as elevated radiation effectiveness, extreme gain, adjustable structure, and exciting mechanisms, dielectric resonators have become a viable alternative for MIMO antennas in recent years [1]. Only a limited investigation articles on MIMO DRA are available in the open literature [2,3,4,5,6]. A rectangular MIMO DRA for 4G applications has been proposed in ref. [2]. However, using a short brooch and a cupper feed complicates the construction. For MIMO applications, Nasir et al. suggested a rectangular DRA [3]. through engraving cuts into the base plane, the separation among the ports has been increased. However, putting an RDRA in an FR4 substratum complicates the manufacturing process. For 5G NR band applications, the current authors proposed a cylindrical MIMO dielectric resonator antenna (CDRA) [4]. The isolation between the ports has been increased by activating the orthogonal mode in the CDRA. MIMO antenna based on DRA for radio access points has been offered in ref. [5]. The desired MIMO antenna can shelter the 2.45 and 5.8 GHz WLAN/5G NR groups with just one group of large and small DRAs.

A MIMO RDRA for 5G NR band applications was discussed in ref. [6]. However, all of the MIMO antennas mentioned above have only shown linear polarization (LP). In recent years, antenna designers have become increasingly interested in using circularly polarized (CP) radiators in MIMO system antennas. Because circularly polarized (CP) antennas are preferred over LP antennas in wireless communication as they provide a consistent connection among the transmission and receipt systems irrespective of their positioning, ref. [7] demonstrates that, rather than employing an LP radiator, using a circularly Polarized MIMO antenna system appears to be a favorable technique in enclosed and outside conditions.

During recent years, many studies have focused on decoupling methods, especially in UWB MIMO antennas through various complex, geometrical feed structures [8]. F-shaped stubs [9], metasurfaces using slots [10], and patch antennas [11] have been introduced to block the electromagnetic (EM) or current waves between radiators. However, the issue with all of these techniques is that they are a bit complicated to adopt; secondly, they are LP antennas. This paper proposes a singly fed, two-element, dual-port RDRA for MIMO and 5G NR band applications. The RDRA uses a modified, novel, A-shaped, conformal metal strip to generate CP waves. The conformal metal strip is oriented so that left-hand circular polarization (LHCP) is produced with one port, along with right-hand circular polarization (RHCP) is generated with the other. As a result, the polarization diversity concept is used in the proposed radiator to enhance the separation among the two radiator ports (|S_12_| < −20). Several DGS cuts are employed in the base plane amongst the two antennas to further improve isolation (|S_12_| < −25). The envelope correlation coefficient (ECC) and diversity gain (DG) are both used toward measure MIMO functioning.

The following is the structure of the article: Section 2 provides details of the construction of the proposed antenna and its study. Section 3 covers the simulation results of the designs and optimization process. Section 4 presents the experimental results and diversity performance, and the conclusion is presented in Section 5.

## 2. Antenna Design and Analysis of the Proposed Radiator

**Figure 1:**
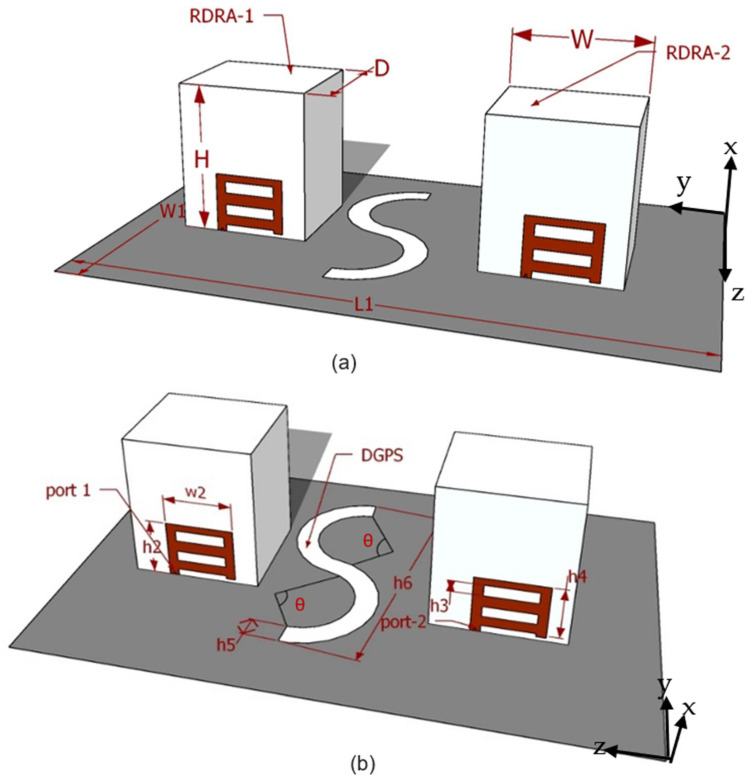
Graphic diagram of the desired MIMO antenna: (**a**) 3D view point; (**b**) feed and DGPS structure.

The proposed MIMO radiator’s feeding mechanism and 3D perspective are depicted in Figure 1a,b. A pair of rectangular DRAs, which were of Eccostock HIK with Ɛr = 9.8 and tanδ = 0.002, were fitted over a metallic ground plane. A novel-shaped, conformal metal strip was installed on the DRA face by the support of adhesive (Quick Fix). At the same time, the radiators were excited through the singly fed mechanism. At ports 1 and 2, both RDRAs, which were mirror images of each other, were utilized. To improve isolation, an S-shaped DGPS slit was etched into the upper half of the base plane among the two radiators. The desired MIMO antenna was supplied by a 50 Ω metalic probes both ports 1 and 2. The optimized parameter for the proposed MIMO RDRA was attained based on rigorous parametric analysis using a finite integration technique (FIT)-based simulation tool, i.e., CST MWS [12], and the values are listed in Table 1.

### Evolution of the RRDA MIMO Antenna

**Figure 2:**
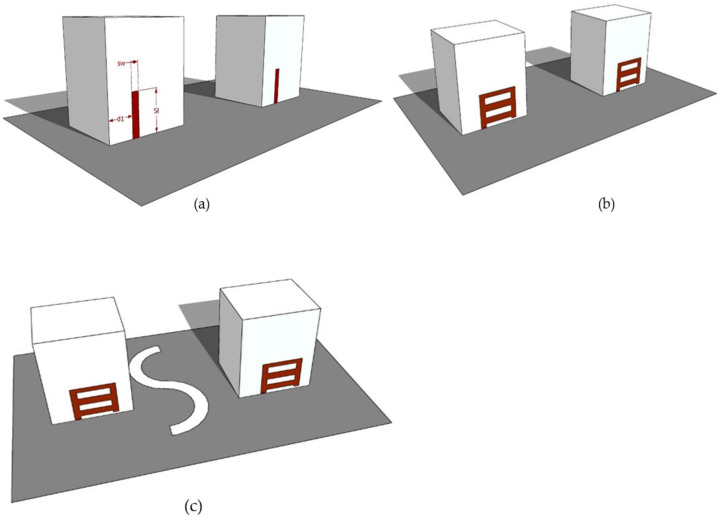
Development of the RDRA MIMO Antenna (3D view): (**a**) Design-1: MIMO RDRA with a single strip; (**b**) Design-2: MIMO RDRA with a novel conformal metal strip; (**c**) desired MIMO RDRA.

Figure 2 illustrates the evolution and building of the intended RDRA antenna. Figure 2a presents the structure that was made up of two rectangular DRs, each of which was fed via a 50 Ω coaxial probe. However, a novel conformal metal strip was used in Step 2, which comprises five individual cut strips. As depicted in Figure 2b, a DGPS was introduced at an optimized location to obtain the desired results.

## 3. Simulation Results and Discussion

In this segment, the optimization, development, and model outcomes of all of the designs are discussed in detail. Section 3.1 focuses on the design and optimization of a LP DRA at its full potential return loss bandwidth along with a single conformal copper strip. Likewise, out of shifting the single copper feed to a novel conformal-metal-strip-shaped feed with optimized parameters excites the orthogonal waves for generating circular polarization. In Section 3.2, to provide improved isolation, we discuss how a DGPS with optimized parameters was introduced at an optimized location, i.e., between the two radiators.

### 3.1. Linearly Polarized MIMO RDRA (Design-1)

The preliminary prototype was intended and evaluated utilizing the DWM model [13]. Through putting the DRA MIMO antenna on a base plane, similar modes were short-circuited, and just odd modes might be present [14]. Since this, odd modes, i.e., TE modes were energized. In corresponding, the operating frequency of the primary sort, $TE_{\delta 13,\;}^{x}$, was calculated employing equations [15] where *k_x_*, *k_y_*, and *k_z_* respectively represent the wave numbers in the *x*, *y*, and *z* routes within the DRA, as indicated in Equation (1):

(1) $$f_{0}=\frac{c}{2\pi \surd Ɛr}\sqrt{k_{x}^{2}+k_{y}^{2}+k_{z}^{2}}$$

(2) $$k_{x}=\frac{\pi }{c}\;$$

(3) $$k_{z}=\frac{\pi }{2H}$$

Likewise, the AR was characterized by:

(4) $$\frac{E_{y}}{E_{x}}=\frac{\left|E_{y}\right|}{\left|E_{x}\right|}\;*\;e^{jҨ}$$

The electrical field ground vector’s y-directed element was *Ey*, even though the x-guided component was *Ex*. For the difference in phase among *Ex* and *Ey*, the breadth ratio was 1, and the phase variation (ɸ) was 90° meant for the pure cp specification performance. The individual strip RDRA (Figure 2a) could have achieved a broader S_11_ bandwidth, but it figure out do not get a axial ratio mode.

Table 2 shows the optimal parameters for the RDRA’s single copper metal feeds, established on meticulous parametric evaluation using the CST. This showed the impedance-matching bandwidth, which was susceptible to feed height, as altering it could degrade the results. After running the parametric sweep, the strip dimensions were optimized, and the optimal measurements of the particular strips were Sw = 11.75 mm and Sw = 1 mm. S11 bandwidths of ~7.19% were achieved with the optimized feed parameters.

**Figure 3:**
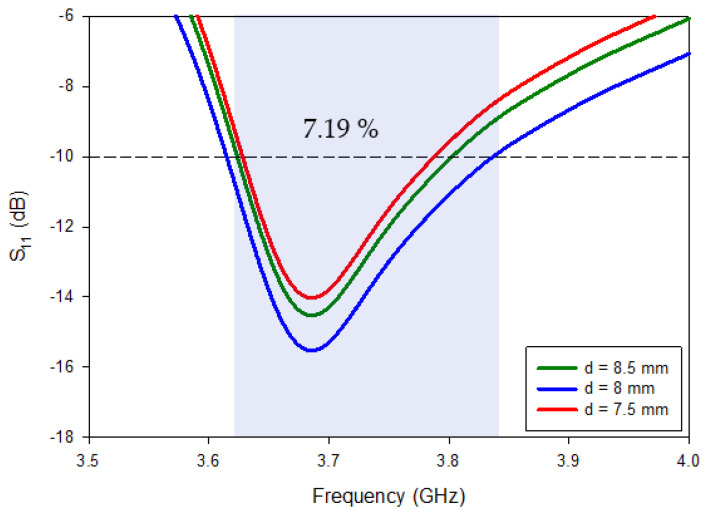
S_11_ of the RDRA feed at various distances from the edge.

Adjusting the feed’s position could manage and optimize the RDRA’s impedance-matching bandwidth (S11) [16]. As a result, parametric research was carried out for various d1 values. The best value of the d1 from the edge is shown in Figure 2a, whereas Figure 3 illustrates the numerous return-loss qualities of the RDRA with various values of d1: 7.50 mm, 8.00 mm, and 8.50 mm as of the side. Every estimate of d1 yielded a various return-loss estimate. The resonant frequency rose (higher frequencies) when the value changed, and the greatest reflection coefficient was attained when the gap was kept at d1 = 8 mm.

**Figure 4:**
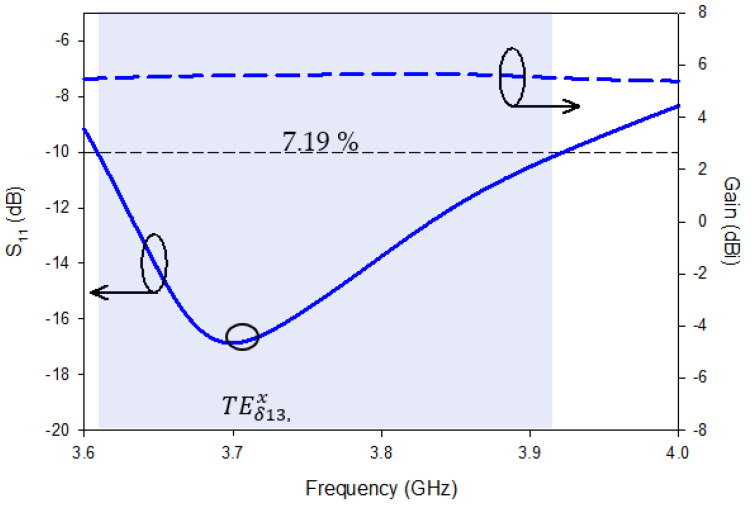
Simulated S_11_ and gain of Design-1.

Thus far away, the improved strips limitations and feed position had been confirmed. The individual metal strip of the DRA provided a computer-generated return-loss (S11) bandwidth of ~7.19%. A desired gain of ~5.2 dBi occurred through out the desired frequency range, as demonstrated in Figure 4; moreover, it is worth mentioning that Design-1 could not produce circularly polarized waves because ports 1 and 2 could excite only a single, higher-order mode $TE_{\delta 13,\;}^{x}$ inside the DR, whereas the generation of CP waves requires the exciting of two orthogonal-mode pairs, with a quadrature phase-shift between them [17]. Consequently, the said design is LP, and to excite the DRA strip, a separate-edge port was applied.

**Figure 5:**
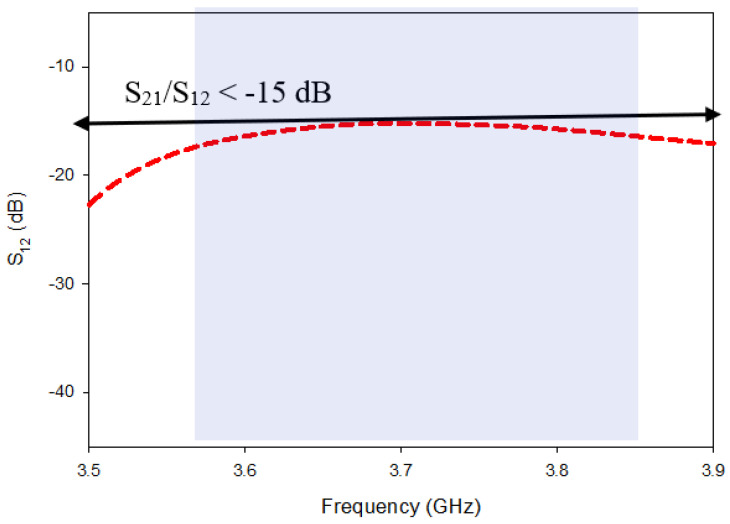
Mutual coupling of Design-1.

Design-1 provided a narrow impedance-matching bandwidth of 7.19% while in parallel, high mutual-coupling, as illustrated in Figure 5. The band achieved via Design-1 covers a range from ~3.61 GHz to 3.95 GHz, having return losses for S_11_/S_22_ of < −10 dB and for S_21_/S_12_ of < −15 dB. Design-1 had only one minima (resonance) in the S11 curve at 3.68 GHz due to the main radiator.

The simulated results of Design-1 were undesirable for a MIMO system; for the best performance, the design of the linearly polarized DRA must be modified to generate circular polarization and widen the S11 bandwidth along with that reducing shared coupling. Modifications with in the design are discussed with in the subsequent subsection.

### 3.2. Design and Optimization of Design-2

Following that, the second configuration of dielectric resonators (DR) was constructed and termed design-2, as illustrated in Figure 2b. This section primarily focuses on the formation of circular polarization and the augmentation of bandwidth, as circular polarization is generated by activating two orthogonal modes within the DRA.s. To that end, design-2 is proposed, in which a novel conformal metal strip is responsible for the excitation of two virtually, orthogonal mode pairs. The antenna array is configured in the H-plane with a corner to corner distance (d) of 18 mm, equating towards o /2 at 3.72 GHz. [18].

**Figure 6:**
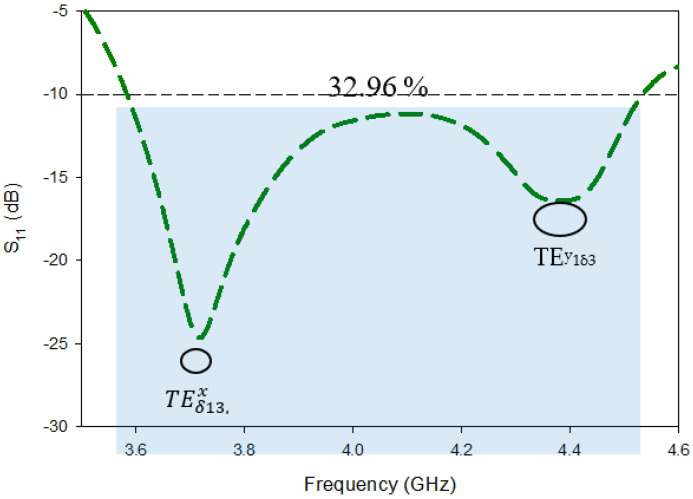
Impedance-matching bandwidth of Design-2.

Because of the multiple sweeps, the components of the revolutionary feed strips have been optimized; the optimal dimensions of the individual strips are h2 = 12 mm, h3 = 2 mm, h4 = 10.5 mm, and w2=10 mm. All of the strips have a width of 1 mm. The aspect ratios and permittivity of the DRA also have an effect on S11 bandwidth. Furthermore, the resonance frequency can be impacted by a number of parameters, including the efficiency of field mutual coupling between the strips and the DRA, the capacity, and the strip’s location. The design approach for the Novel-shaped conformal metal strip is summarized in Table 3. Incorporating all of the strips at the optimal location and characteristics results in produced an additional resonant frequency at ~4.37 GHz, which, in parallel, widened the S11 bandwidth from 7.19% to 32.96%. The S11 band covered by Design-2 ranged from 3.59 GHz to 4.55 GHz; because of this, it may be used for sub-6 GHz 5G applications. Impedance-matching bandwidth of Design-2 is depicted in Figure 6.

### 3.3. Generation of CP Waves

Because of the new feed shape and symmetrical structure, it is possible to generate circular polarization and increase bandwidth. Few studies on the similar idea of increasing S11 bandwidth and generating CP BY employing symmetrical designs have been published [19,20,21]. By improving the sizes, two smidgins S11 points that vibrate at close frequencies can be generated. As a result, two degenerate modes are formed, and because they are orthogonal to one another, they produce C.P. The degenerate mode pair TEx13 at 3.85 GHz and TEx13 at 4.5 GHz were thrilled to generate the Cp wave. For the creation of the C.P signals, the initial criterion of orthogonal modes is met. Furthermore, design-2 has least C.P, or 3.90 GHz, separating the degenerated modes. As a result, both prerequisites for C.P generation [22] and [23] are met. 

**Figure 7:**
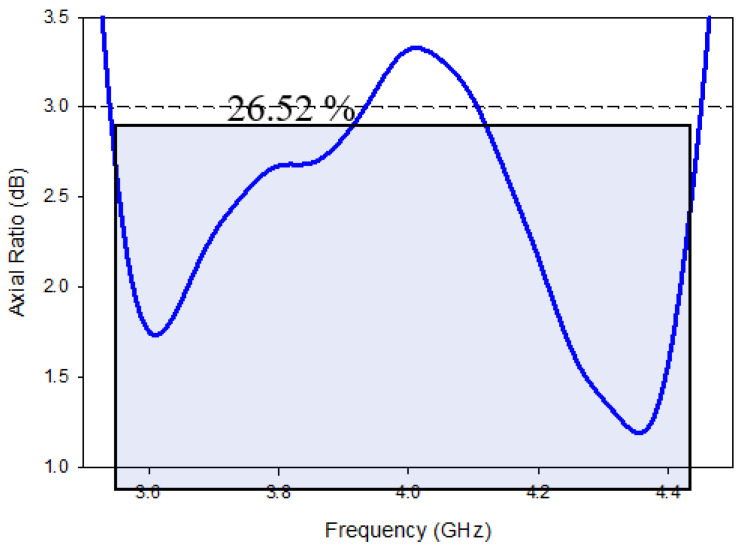
Axial ratio bandwidth of Design-2.

The simulated 3-dB axial ratio is shown in Figure 7 after all of the settings have been optimized. In addition to an return loss bandwidth over the desired frequency range, the proposed excitation provided simulated cp concluded a bandwidth of about 26.52 percent (3.55 GHz–4.45 GHz). TEx13 and TEy13 simulated mode frequencies, at 3.8 GHz and 4.53 GHz, respectively, are extremely close to those anticipated by the DWM [24].

**Figure 8:**
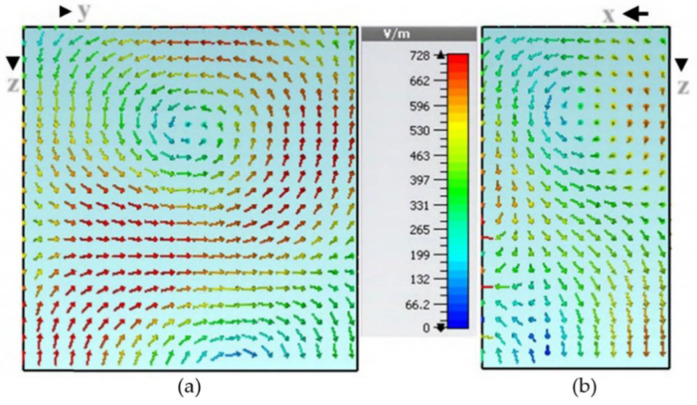
Division of electric field (**a**) TE_x_^δ13^ at 3.74 GHz and (**b**) TE_y_^1δ3^ at 4.4 GHz.

**Figure 9:**
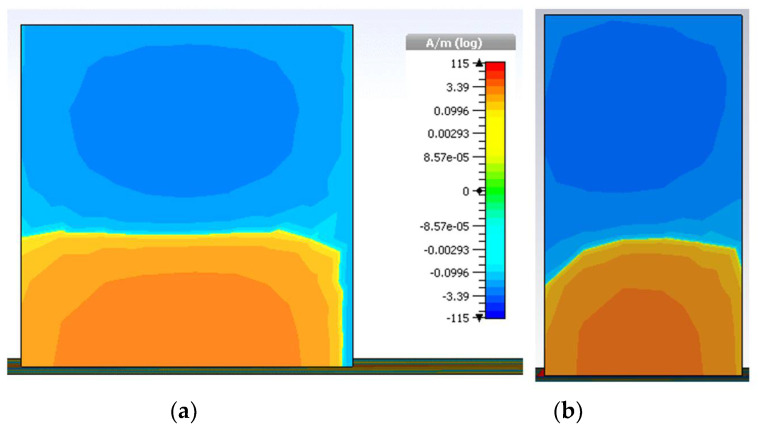
Distribution of magnetic field (**a**) TE_x_^δ13^ at 3.72 GHz and (**b**) TE_y_^1δ3^ at 4.35 GHz.

The simulated electric field and magnetic field distributions are illustrated in Figure 8 and Figure 9, respectively, to justify the degeneration of two orthogonal modes.

### 3.4. Mutual-Coupling Reduction

Mutual coupling is the amount of energy realized by a nearby antenna when another antenna broadcasts. Mutual coupling alters the reflection coefficient, radiation pattern and input return loss of RDRA MIMO antennas. [25,26] are the basic mutual coupling, *MC ij* empirical models.

(5) $$MC\;ij\;=\;\exp\;\left(-\frac{2d_{ij}}{\lambda }\left(\alpha +j\pi \right)\right).i\;\neq j$$

(6) $$\mathrm{MC}\;ij\;=\;1\;-\;\frac{1}{N}\sum_{I}\sum_{i\;\neq j}d\;ij$$

Somewhere *dij* is the space among the ith and jth antenna elements, is the mutual coupling intensity regulating parameter, and N is the quantity of array components. Mutual coupling in practice is determined by the array configuration and the energizing of other components. It is often expressed in dB-values, S-parameters among the ith and jth antenna elements, and 20log10(Sij) separation. However, the precise mechanism of reciprocal coupling is mostly determined by the transmitting and receiving modes [27,28].

**Figure 10:**
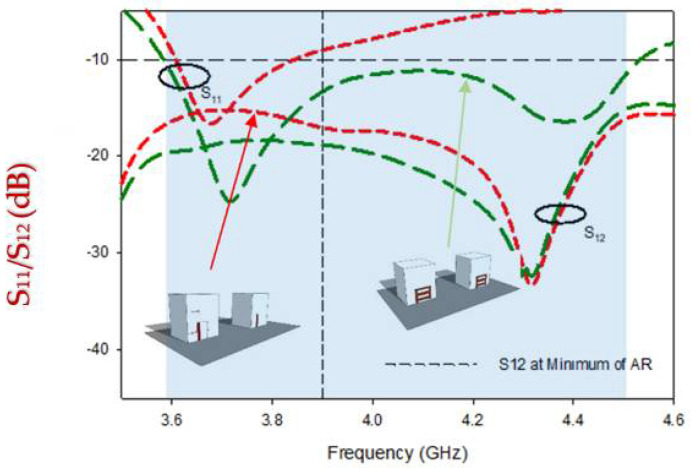
Comparison of mutual coupling in Design-1 and Design-2.

Despite the fact that Design-2 generates Circular polarization and broadens the impedance matching bandwidth, the major parameter, Mutual coupling (S12/S21) between the radiating parts, is reduced by a little, i.e., −19 dB, whereas Design-1 was −15 dB. In Figure 10, the modeled S-parameters of Design-1 and Design-2 are indicated for comparison. The minimum isolation level of Design-1 is -16 dB at 3.69 GHz in the S11 band (3.62 GHz–3.84 GHz), while isolation improves to −19.9 dB at the same frequency in Design-2. Mutual coupling remains significant at lower frequencies (3.5 GHz–4. GHz), but steadily reduces as frequency increases, with the lowest coupling obtained at 4.3 GHz for both Design-1 and Design-2.

### 3.5. The Design and Optimization of the Proposed Design

In Design-1 and Design-2, the DRA elements were placed side by side, and the feeding was executed from one end of the PEC ground plane. Although broadband S11 and 3 dB axial ratio was attained through Design-2, in parallel, this configuration did not significantly bring mutual coupling (MC) down. Hence, the main focus of this step was to reduce MC. At higher frequencies, MC is reduced by optimizing the spacing between two DRA elements. However, the tightly spaced DRA configuration led to high mutual coupling at a low-frequency range of 3.5–5 GHz. This high mutual coupling at a low-frequency range was reduced by rearranging the DRA elements and feeding network, as shown in Design-2. Finally, to further reduce mutual coupling toa level below −19 dB for the entire band of interest, an S-shaped DGPS was introduced in the ground plane.

The effect of the distance between the DR element on the S-parameters and the far-field parameters is shown in Table 4. The table presents the S11 bandwidth, mutual coupling reduction, 3-dB AR, return loss, and the fraction of intersecting bandwidths. The outcomes demonstrate that the 3-dB radiation bandwidth and isolation were easily enhanced throughout the range from 0.05 λ to 0.55 λ, i.e., 4 mm–15.00 mm, that is a preferred element in a MIMO DRA design. On the other side, there was a noteworthy difference in the gain for the various lengths. On the basis of these results, the desired distance for the proposed design was 0.15 λ (12.15 mm), where λ0 was the wavelength concerning the lowest frequency of operation.

**Figure 11:**
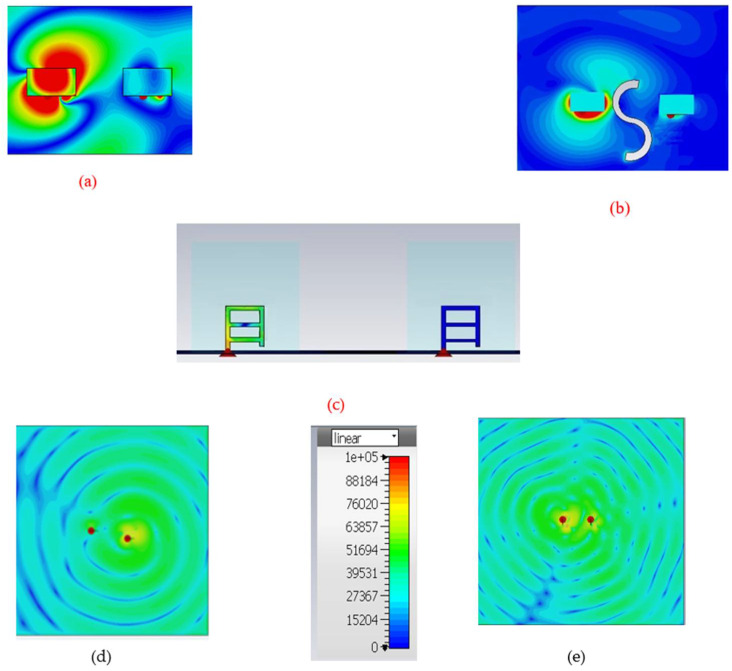
(**a**) Surface wave current before deputing DGPS (top view) at 3.89 GHz; (**b**) Surface wave current after deputing DGPS at 3.89 GHz (top view); (**c**) Surface wave current after deputing DGPS at 3.89 GHz (front view); (**d**) Surface wave current on the ground plane before deputing DGPS at 3.89 GHz (counterclockwise); and (**e**) Surface wave current on the ground after deputing DGPS at 3.89 GHz (co-centric).

Mutual coupling between antennas occurs also due to spreading surface waves or through radiation; it is evident from Figure 11 that, before deputing DGPS, the density of surface current was high on the entire DR. Consequently, after placing the DGPS at an optimized position and angle, a much smaller sum of current was connected to the another antenna, and because of this, the isolation between the ports was reduced significantly. In all of the above cases, port 1 was excited, which is on the left side of the configurations. Also, the surface wave-current changes from a counter-clockwise to a co-centric motion because of DGPS. This improvement in isolation between the two antennas could be easily explained with the help of surface current distribution at a minimum of an axial ratio of 3.89 GHz.

**Figure 12:**
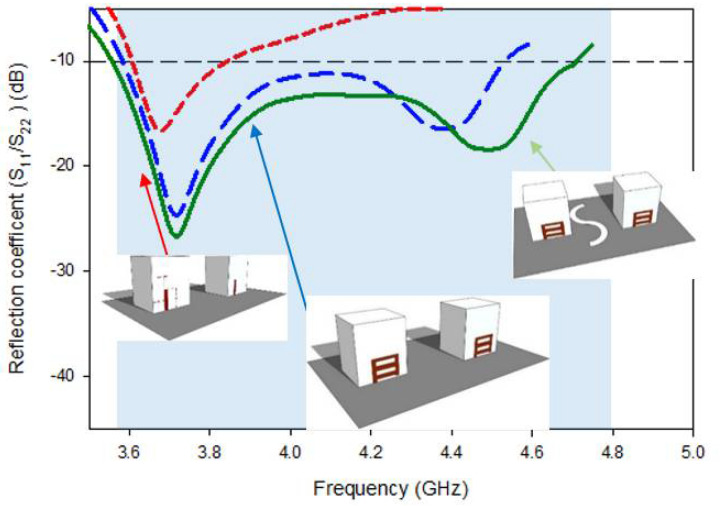
Comparison between the return loss of Design-1, Design-2, and the proposed design.

The simulated return-loss act of the desired MIMO RDRA is depicted in Figure 12. It is clear from the first resonant frequency remains the same as compared to that of Design-2, while the second resonance frequency marginally moved from 4.4 GHz to 4.5 GHz due to the new placement of the radiating element at a 0.15λ position which, as a result, enhanced the bandwidth a bit. Simulated analyzed conclusions disclosed that the proposed DRDA MIMO had a return loss of 24.19%, covering the range from 3.57 GHz to 4.68 GHz. The attained S11 bandwidth was about 2.62% more than the bandwidth of Design-2. The previous section explains the two resonance modes in the S11 results because of the novel conformal metal strips.

**Figure 13:**
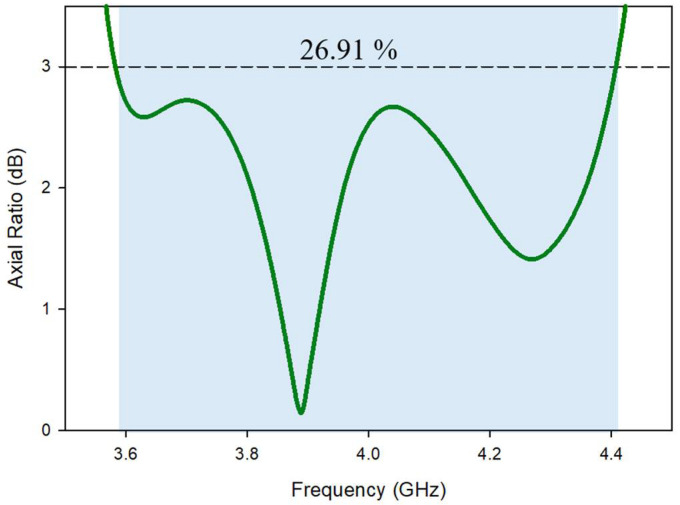
Axial ratio of the proposed antenna.

On the other hand, Figure 13 illustrates the CP bandwidth of the proposed design. It has been found that the inclusion of the DGPS has not such a considerable impact on the CP ratio. The simulated circular polarization bandwidth (3 dB) achieved by the proposed design was 22%, which was achieved over the band from ~3.55 GHz to 4.40 GHz. The minimum of axial ratio was achieved at 3.88 GHz. The obtained AR bandwidth of 100% occurred in conjunction with impedance-matching bandwidth.

**Figure 14:**
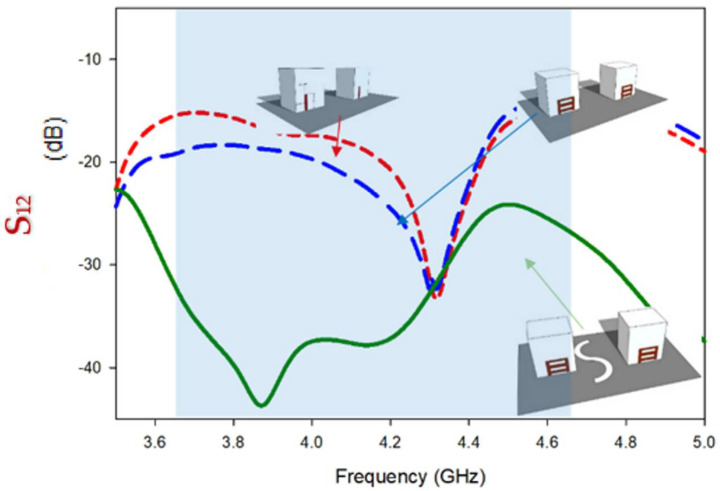
Comparison of mutual coupling reduction in Design-1, Design-2, and the proposed design.

By placing the DGPS and radiating elements at their optimum positions, mutual coupling was reduced significantly, as illustrated in Figure 14; while explained earlier; mutual coupling occurs either through radiation or surface currents on the ground; by placing both DRs in the optimum position, at 0.15 λ, with the working mechanism of the DGPS, these steps overcame the issues which were experienced in Design-1and Design-2. High MC in the low-frequency band is due to the surface waves coupling between feed elements [29], and based on the simulated result, as depicted in the results, suitable isolation of more than −28 dB was achieved on average (12 dB at 3.65 GHz, 26 dB at 3.89 GHz, 16 dB at 4.85 GHz).

Furthermore, Table 5 compares the performances of the proposed antenna with respect to the previous work based on the technique for reducing MC, return loss bandwidth, and the type of antenna. In parallel, the proposed antenna is also compared with other circularly polarized MIMO antennas discussed in the literature. The comparisons are presented in Table 6.

## 4. Fabrication and Experimental Verification

Fabrication and testing of a single-fed RDRA MIMO antenna using a single feed mechanism are carried out. Al_2_O_3_ ceramic material Ɛr = 9.8 was used to make the dielectric resonator, which was subsequently placed on the PEC ground plane. in a microwave, chamber to quantify scattering parameters (|S11| measurement), and the EMT-24 SATIMO measuring system was used to calculate other parameters, such as axial ratio, pattern radiation, and realized gain. Traditional feeds (for example, probe connector, Coaxial probe feed, and microstrip line) consume extra downsides as compared to conformal copper feeds, because of this e can be used instead. Air breaks, which produce frequency discrepancies, are difficult with coaxial feedings. Conformal strips, In parallel, can be easily attached to the SMA connectors [30]. Conformal feeds help reduce air gaps because it is comprised of adhesive glue copper (Cu) duct tape that attaches to the MIMO RDRA side swiftly and securely.

### Return Loss

**Figure 15:**
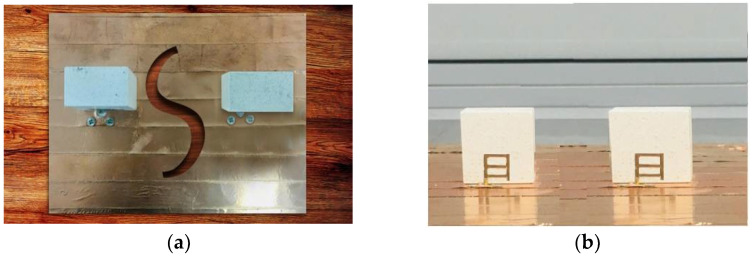
Photographs of a fabricated, circularly polarized, wideband MIMO antenna: (**a**) Top view with DGPS; (**b**) Front view without DGPS.

The suggested antenna has a wide impedance BW S11/S2 coverage of 37.62 percent throughout the entire frequency range of 3.6–4.84 GHz. Over the whole band, the measured MC S21/S12-28 dB is reached. The agreement between simulation and measurement is good, and the tiny gap is primarily due to experimental defects and fabrication mistakes. The simu-lated and measured graphs show two modes, which are caused by a new conformal metal strip introduced at an optimal distance. where Figure 15a depicts the top view of the fabricated antenna. Where Figure 15b shows the front view with DGPS.

**Figure 16:**
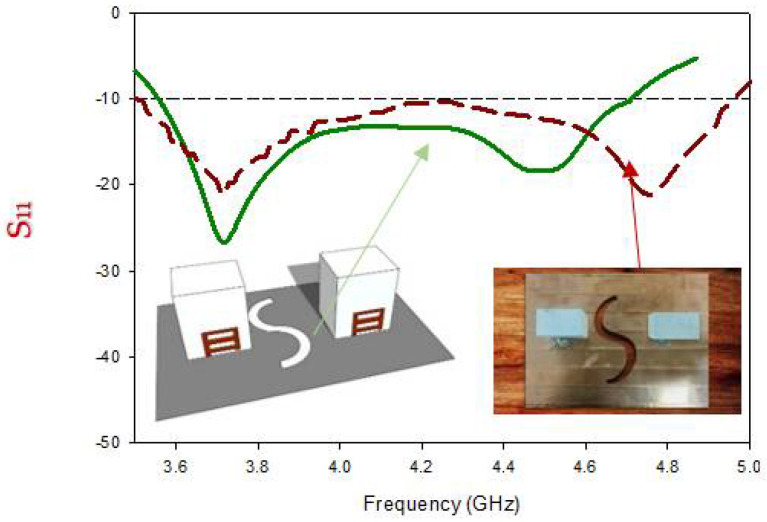
Comparison of the return loss of the fabricated and simulated MIMO antenna.

Figure 16 depicts a comparison of the proposed MIMO antenna’s calculated and simulated S-parameters. The reflection coefficient bandwidth is to be computed as 37.14 percent, that is a bit greater as compared to the fabricated values of 36.63 percent. with a minor difference due to the use of a predetermined PEC plane and fabricated acceptance Furthermore, due to the small mass of the antenna, it was impossible to completely reduce the possible air holes. There is a 1.55 percent variation among the two pairs of results because the lowest S11 is calculated at 3.73 GHz against 3.72 GHz in the fabrications. Because of this, the findings accord nicely with the expected operating frequency for the mode induced by the DRA computed using the DWM approach. The comparison of the tested and simulated reflection coefficients shows that the tested findings closely match the simulated results.

**Figure 17:**
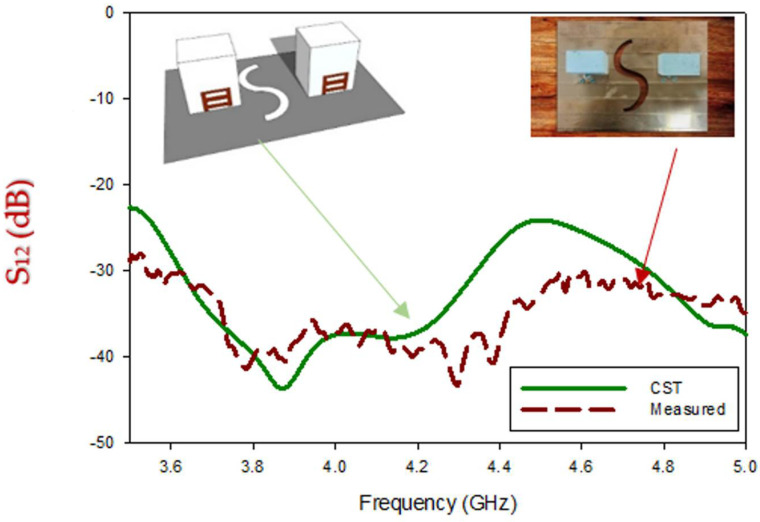
Comparison of the S_12_ of the fabricated and simulated MIMO antenna.

With reference to Figure 17, it is noticed that theoretical results of the transmission coefficient of wideband circularly polarized MIMO antenna depict suitable isolation (S_12_/S_21_ ≤ −28 dB) through the circularly polarized operating band. During the measurement, one port is excited while one is attached to a 50 Ω paired load. The measured isolation is varied with respect to frequency. Maximum Measured isolation between the radiators is achieved at 4.3 GHz. On the other side, Isolation reduces gradually from 4.3 GHz to 4.65 GHz, and the minimum isolation is attained at ~4.65 GHz.

**Figure 18:**
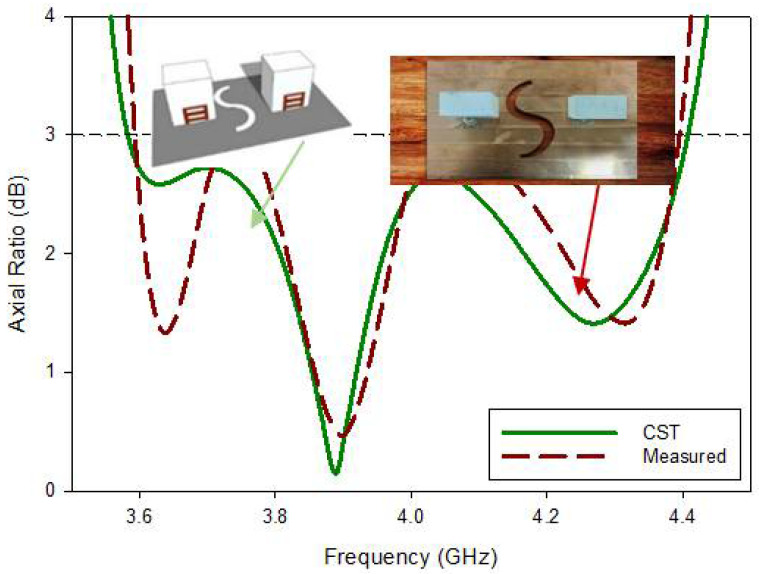
Comparison of the axial ratio of the fabricated and simulated MIMO antenna.

The axial ratio broad bandwidth was calculated and evaluated in the weary-sight track, as presented in Figure 18, as its clear that the least calculated AR is 0.35 dB at 3.89 GHz, equated to a comparable assessed quantity of 0.55 dB at 3.91 GHz. With orientation book to the shape, the simulated cp bandwidth extended on or after 3.60 to 4.4. The GHz was 3.61, as compared to 4.39 GHz in the measurements. Furthermore, based on the findings, the intersecting 3-dB AR and return loss bandwidths from the simulated and fabricated were 100%. The theoretic efficient 3 dB AR bandwidth was found to be 28.33%, compared to 26.52% in the experiment. It was observed that the entire axial ratio bandwidth completely fell within the S_11_ bandwidth.

**Figure 19:**
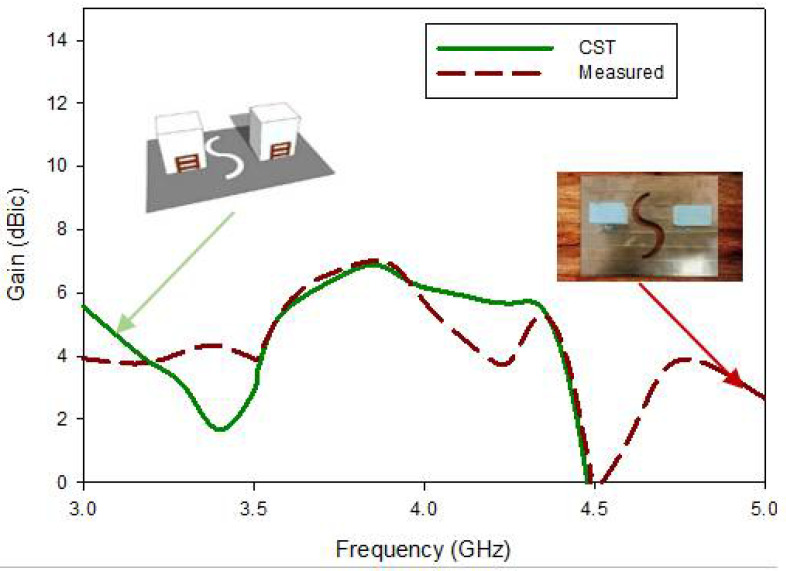
Comparison of the gain of the fabricated and simulated MIMO antenna.

After measuring the near-field parameters, we turned to the measurement of the far-field parameters, which started with measuring gain with the help of the substitution method, that is, having two standard horn antennas and the AUT (proposed antenna). The simulated gain of the proposed 1 × 2 CP MIMO RDRA antenna at a minimum axial ratio (3.88 GHz) was 6.01 dBic. Correspondingly, for the designed 1 × 2 CP wideband MIMO, the measured value of gain at the minimum of the resonance frequency of the axial was ~6.0 dBic. The theoretical and measured average gain readings are depicted in Figure 19. over the entire frequency band of interest. An average gain of ~6.00 dBic has been attained through the cp bandwidth in the simulated and fabricated results.

Similarly, the behavior of the desired MIMO RDRA antenna demonstrates through ECC and DG. Information regarding the coupling at the ports illustrates only through isolation parameters (*S*_12_/*S*_21_).

On the other hand, ECC comprises of all the scattering parameters of the proposed wideband circularly polarized MIMO DRA in order to demonstration their effects on the correlation coefficient. If the value of ECC is Low its means have less correlation among radiating elements; however, having higher values of ECC is not considered to suitable for MIMO applications for a decent level of performance. By using the formula shown in Equation (7), the values of the ECC can be obtained

(7) $$\rho_{e}=\frac{{\left|S_{11}S_{12}+S_{21}S_{22}\right|}^{2}}{\left(1-{\left|S_{11}\right|}^{2}-{\left|S_{21}\right|}^{2}\right)\left(1-{\left|S_{22}\right|}^{2}-{\left|S_{12}\right|}^{2}\right)}$$

**Figure 20:**
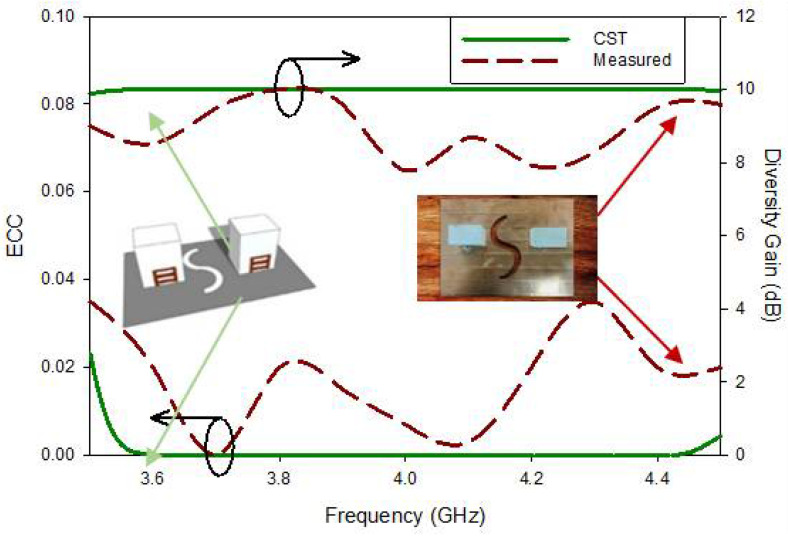
ECC and DG of the proposed MIMO antenna.

The theoretical values of the ECC lie in the range of 0–0.02, as depicted in Figure 20, throughout the whole frequency band, while in the same frequency band, the experimental values were between 0–0.36 between the two radiators. At minimum of axial ratio (3.88 GHz) the simulated value of ECC is 1.37 × 10^−6^, and in parallel experimental value of ECC at same frequency is 0.02. By exploitation far-field radiation samples can help to determine ECC along with the assistance of Equation (8) [31]

(8) $$\rho_{e}=\frac{{\left|\iint_{4\pi }^{}\left[{\vec{F}}_{1}\left(ɵ,ɸ\right).{\vec{F}}_{2}\left(ɵ,ɸ\right)\right]d\Omega \right|}^{2}}{\iint_{4\pi }^{}{\left|{\vec{F}}_{1}\left(ɵ,ɸ\right)\right|}^{2}d\Omega \iint_{4\pi }^{}{\left|{\vec{F}}_{2}\left(\Theta ,ɸ\right)\right|}^{2}d\Omega }$$

DG is an additional measure utilized to test the implementation of a MIMO antenna (DG). The ideal DG value is 10, although anything greater than 6 is considered desirable in reality, and the measured average DG The suggested broadband CP- RDRA MIMO antenna has a value more than 8.5 dB, as illustrated in Figure 20. The subsequent formula be utilized to compute the defected ground of a desired antenna [31].

(9) $$\mathrm{DG}=10\sqrt{(1-\rho_{e}{}^{2}}$$

## 5. Conclusions

The separation among the DR’s was increased as a result of announcing the DGPS at the ideal position of the DRAs in this paper, which studied a wideband circularly polarised DRA-MIMO antenna. The innovative conformal metal strip enabled CP, which resulted in the degeneration of two orthogonal modes as well as an increase in impedance matching BW. (TE (13)x, and TEy13). The findings for ECC and DG, which are critical diversity performance factors for MIMO antennas, are proven to be within acceptable bounds. Furthermore, the proposed antenna has been constructed and tested, and the calculated scattering values closely match the measured scattering parameters. The antenna has the potential to be beneficial in 5G NR applications.

## Figures and Tables

**Table 1:** Optimized values of the proposed MIMO antenna’s parameters.

Element	Parameter	Dimension (mm)
PEC Ground Plane	L_1_, W_1_	50, 50
RDRA	H, W, D	26, 25, 20
DGPS, Arc	h_5_, h_6_, θ	2.5, 30, 80°
Conformal Metal Strip	h_2_, h_3_, h_4_, w_2_	12, 2, 10.5, 10

**Table 2:** Optimisation of the single-strip measurements in mm.

Sl	Sw	10-dB S11 Bandwidth (%)
10.75	0.5	5.43
		5.18
	0.75	5.92
		4.65
		4.92
		5.17
		5.42
		5.96
		6.23
		6.58
11.75	1	6.89
		7.19
		7.01
		6.75
		6.51
		4.90
12.75	1.75	4.42
		4.20
		3.97
		3.75
		3.54
	2	3.34
		5.91

**Table 3:** Optimization of the Novel conformal strips (unit: mm).

H2 (mm)	H3 (mm)	H4 (mm)	W2 (mm)	Width of Strip (mm)	10-dB S11 Bandwidth (%)
11	1	7	0	1	22.43
		7.5	1	1	22.18
	1.5	8	2	1	23.92
				1	23.65
				1	23.40
		8.5	3	1	24.14
				1	25.67
				1	25.42
		9		1	26.16
			4	1	27.63
12	2			1	28.38
		10.5		1	31.89
			5	1	32.97
				11	32.38
		11		1	29.13
		11.25	5.50	1	28.51
13	3	11.50	5.75	1	28.90
		12	6	1	26.42
		13	7	1	26.20
			8	1	23.97
		3.54	9	1	23.75
	3.5	15	10	1	23.54
				1	22.34

**Table 4:** S11, AR bandwidth, gain, and isolation at different spacings.

Distance (mm)	Return Loss (S_11_) %	Axial Ratio (3 dB) %	Gain dBi (dBi)	Mutual Coupling (dB)
0.05 λ (4)	35.34	nil	5.8	nil
0.10 λ (8)	36.26	25.74	5.33	−22.7
0.15 λ (12)	**36.63**	**26.52**	**6.51**	**−28.40**
0.20 λ (16)	35.69	25.53	5.60	−26.24
0.25 λ (20)	34.05	25.59	5.80	−23.45
0.30 λ (24)	34.76	24.59	5.89	−24.10
0.35 λ (28)	32.39	24.62	5.95	−24.85
0.40 λ (32)	32.20	24.87	6.09	−25.17
0.45 λ (36)	31.05	25.47	6.10	−26.14
0.50 λ (40)	30.12	23.44	6.51	−26.40
0.55 λ (44)	29.22	22.24	6.00	−27.14

**Table 5:** Comparisons with other MIMO antennas.

Literature	Shape of the Radiating Elements	Isolation Technique	BW (GHz)	Isolation (dB)
Ref [6]	Fractal DRAs	DGS and sliding	3.89–10.4	~15
Ref [10]	Fractal DRAs	DGS and sliding	8.36–12.6	~18
Ref [21]	Cylindrical DRA	SRR and Meta-surface Shield	56.6–64.5	~28
Ref [22]	Rectangular DRA	MPR	5.10–5.80	~16
Ref [6]	Rectangular DRA	Sliding	3.4–4.40	~24
Proposed Antenna	Rectangular DRA	DGS	3.35–4.5	~28.25

**Table 6:** Comparisons with other MIMO antennas on the basis of CP.

Literature	Antenna Type	Isolation Technique	3 dB BW (%)	Isolation (dB)
Ref [27]	DRAs	DGS and sliding	13.23	~17
Ref [28]	DRAs	DGS and sliding	3.50	~19
Ref [29]	Hybrid	SRR and Metasurface Shield	3.55	~31
Ref [30]	Printed	MPR.	1.03	~18
Ref [16]	DRA	Sliding and Parasitic Patch	26	~24
Proposed Antenna	Rectangular DRA	DGS	28.33	~28.25

## Data Availability

All data has been included in study.
